# Assessing the Impact of Water Filters and Improved Cook Stoves on Drinking Water Quality and Household Air Pollution: A Randomised Controlled Trial in Rwanda

**DOI:** 10.1371/journal.pone.0091011

**Published:** 2014-03-10

**Authors:** Ghislaine Rosa, Fiona Majorin, Sophie Boisson, Christina Barstow, Michael Johnson, Miles Kirby, Fidele Ngabo, Evan Thomas, Thomas Clasen

**Affiliations:** 1 Department of Disease Control, London School of Hygiene and Tropical Medicine, London, United Kingdom; 2 Department of Civil, Environmental and Architectural Engineering, University of Colorado at Boulder, Boulder, Colorado, United States of America; 3 Berkeley Air Monitoring Group, Berkeley, California, United States of America; 4 Ministry of Health, Government of Rwanda, Kigali, Rwanda; 5 Department of Mechanical and Materials Engineering, Portland State University, Portland, Oregon, United States of America; 6 Department of Environmental Health, Emory University, Atlanta, Georgia, United States of America; Cardiff University, United Kingdom

## Abstract

Diarrhoea and respiratory infections remain the biggest killers of children under 5 years in developing countries. We conducted a 5-month household randomised controlled trial among 566 households in rural Rwanda to assess uptake, compliance and impact on environmental exposures of a combined intervention delivering high-performance water filters and improved stoves for free. Compliance was measured monthly by self-report and spot-check observations. Semi-continuous 24-h PM_2.5_ monitoring of the cooking area was conducted in a random subsample of 121 households to assess household air pollution, while samples of drinking water from all households were collected monthly to assess the levels of thermotolerant coliforms. Adoption was generally high, with most householders reporting the filters as their primary source of drinking water and the intervention stoves as their primary cooking stove. However, some householders continued to drink untreated water and most continued to cook on traditional stoves. The intervention was associated with a 97.5% reduction in mean faecal indicator bacteria (Williams means 0.5 *vs*. 20.2 TTC/100 mL, *p*<0.001) and a median reduction of 48% of 24-h PM_2.5_ concentrations in the cooking area (*p* = 0.005). Further studies to increase compliance should be undertaken to better inform large-scale interventions.

Trial registration: Clinicaltrials.gov; NCT01882777; http://clinicaltrials.gov/ct2/results?term=NCT01882777&Search=Search

## Introduction

Environmental contamination at the household level is a major cause of death and disease, particularly among rural populations in low-income countries. Unsafe drinking water, together with poor sanitation, account for an estimated 0.9% of the global burden of disease and 0.3 million deaths [1]. Much of this disease burden is associated with diarrhoea, which alone accounts for 10.5% of deaths in children under 5 years in low-income countries [2]. Household air pollution (HAP) from biomass fuel smoke has been linked to increased risk of respiratory tract infections, low birth weight, exacerbations of inflammatory lung conditions, cardiac events, stroke, eye disease, tuberculosis, cancer and nutritional deficiencies [3]. The Global Burden of Disease (GBD) 2010 project found HAP from solid fuels to be responsible for 3.5 million premature deaths globally [1]. In this same assessment, smoke from household cooking fuels was also responsible for another half a million premature deaths due to contributions to outdoor air pollution [1]. These environmental hazards are aggravated among rural inhabitants of sub-Saharan Africa who are more likely to rely on unsafe water supplies and cook using biomass fuels on inefficient stoves [4]–[6].

Inefficient cookstoves also present substantial economic, developmental and environmental costs. At the household level, poverty is exacerbated and time spent at school is reduced by the burden of collecting more fuel for boiling drinking water and cooking [7]. Individuals, households and governments bear the cost of expenditures for seeking treatment of enteric and respiratory infections. Cookstove emissions also contribute to greenhouse gas and black carbon emissions, and in some cases the fuel harvesting can result in denuding of forests [8], [9].

With a population of 10.5 million and a density of 412 persons per sq. km, Rwanda is the most densely populated country in East Africa [10]. Eighty per cent of the population of Rwanda lives in rural areas and is engaged in agriculture [11]. Despite significant progress over the last decade, 57% of the population is living below the poverty line, 37% of them living in extreme poverty [11]. While a large proportion of the rural population has access to improved water sources (71.2%), mainly through protected springs, only 2.2% of rural areas have water on their premises [12], resulting in an increased risk for drinking water contamination during transport and storage [13]. Almost all of rural Rwanda (99.0%) relies on biomass for their cooking needs [12]. Morbidity and mortality are largely dominated by communicable diseases, including HIV/AIDS, acute respiratory infections, diarrhoeal diseases, intestinal parisitoses, and malaria [14]. Among deaths of children under 5, pneumonia accounts for 20% and diarrhoea for 12% [15].

In an effort to reduce the disease burden in rural Rwanda, decrease poverty associated with expenditures for fuel, and minimize the impact of greenhouse gases from inefficient combustion of biomass in low-efficiency stoves, the Rwanda Ministry of Health (MiniSante) and the Rwanda Environmental Management Authority (REMA) have partnered with DelAgua Health (implementer) to design, deploy and evaluate the impact of a project that will deliver and promote the use of advanced water filters and high efficiency cookstoves to lower-income households in Rwanda. Prior to initiating the full campaign, the implementer with the Ministry of Health undertook a pilot distribution of filters and cookstoves to approximately 2200 households in 15 villages in 11 of the country's 30 districts. We conducted this study in three of those villages in order to assess the uptake of the intervention and its impact on drinking water quality and household air pollution.

## Methods

### Study setting

The study was conducted from September 2012 to April 2013 in three rural villages, Nyarutovu and Kabuga located in Muhanga district, Southern province; and Rubona, located in Gakenke district, Northern province. These villages were purposely selected from the 15 villages comprising the pilot distribution phase. The sites were changed from the original protocol, Karongi and Ngororero districts in Western province, to accommodate access to better microbiology laboratory facilities in Kigali.

### Study design and sample size

The study employed a parallel, household-randomised, control trial design with a 1∶1 ratio. This trial followed a non-blinded design because previous attempts to blind an earlier version of the LifeStraw Family filter in the Democratic Republic of Congo were unsuccessful [16]. The objectives of the study were to assess (i) uptake and use of the intervention by the target population when delivered programmatically, and (ii) the impact of the intervention on the microbiological quality of household drinking water and air quality near the self-reported cooking area over the 5-month follow-up period. Our primary outcomes were (i) to assess levels of faecal contamination (measured by thermotolerant coliforms, TTC) in stored water in the home that householders used for drinking, and (ii) to determine average 24-h concentrations of PM_2.5_ in the main cooking area as identified by participants. Our secondary outcome was to assess use of the intervention filters and stoves based on self-report and spot-check observations.

The sample size calculation was based on PM_2.5_ emissions reductions rather than TTC reductions in drinking water as the former was determined to require a larger sample. Assuming a 50% reduction in PM_2.5_ emissions, 80% power, α = 0.05 and a coefficient of variation (COV) of 1, we estimated a sample size of 63 households per arm.

The protocol of this trial and CONSORT checklist are available as supporting information; see Text S1 and S2.

### Intervention

Each intervention household received one LifeStraw Family 2.0 filter and one EcoZoom Dura improved wood burning stove. The filter is the second-generation of a gravity-based water purifier that uses ultrafiltration in the form of a hollow-fibre cartridge to remove pathogens from drinking water. The first generation device has been shown in field studies to be highly effective in improving water quality and to achieve consistent (though not exclusive) use [16]. The second-generation version used in this study employs a table-top design and an integrated safe storage vessel. Untreated water is poured through a 20-µm pre-filter plastic mesh into a 6.0 L container; over time, gravity forces the water through the cartridge comprised of hollow-fibres with a 20-nm pore size. The water then passes into a 5.5 L storage vessel where it can be dispensed via a plastic tap. The device is cleaned daily by backwashing the cartridge using a squeeze-pump mounted on the back of the storage container. The device is designed to treat 18,000 L of water [17] with a flow rate of approximately 3 L per hour. In the laboratory, the filter cartridge was found to meet the USEPA standards for microbiological water purifiers by reducing bacteria by 6 logs, viruses by 5 logs and protozoa by 4 logs [18]. The filter meets the “highly protective” World Health Organization (WHO) rating for household water treatment technologies [19].

The intervention stove is based on the ‘rocket’ concept that uses an internal ‘chimney’ in the stove that directs air through the burning fuel (usually biomass), and encourages the mixing of gases and flame above it. Precise internal stove dimensions are used to achieve high combustion efficiency and transfer heat to the cooking pot. Two additional components are included with the stove, a “stick support” onto which fuel wood is placed to promote airflow and a “pot skirt” which increases fuel efficiency. A study comparing cookstoves in Uganda, Kenya and Tanzania reported that the EcoZoom (aka StoveTec) stove saved 39% to 54% of fuel compared to open fires, cooked meals faster, and was participants' most preferred stove during controlled cooking of local dishes [20]–[22]. In the intervention group, householders were encouraged to cook outdoors on the EcoZoom stove and to use dry wood only to increase the efficiency of the stove. Further details on the messaging used in the pilot distribution can be found elsewhere [23].

Houses that were allocated to the intervention group also received a poster with illustrations and instructions in *Kinyarwanda*, the local language, on filter and stove use, maintenance, and contact names and phone numbers for the implementer. Most households had easy access to a cellular phone for contacting the implementer. Intervention households received one-to-one training on use and maintenance in their homes by community health workers (CHWs) who were previously trained by trainers who themselves had been trained by the filter and stove manufacturers and implementer. Intervention households were then visited periodically at approximately one-month intervals by CHWs to refresh health messaging and encourage use. Households allocated to the control group were instructed to continue usual practices throughout the study. At the end of the study in April 2013, these control households received their own filters, stoves, posters and training.

### Enrolment, baseline survey, randomisation and deployment of devices

Households were eligible to participate in the study if (i) they were registered as being members of the village, (ii) the head of the household was over 18 years, and (iii) no members of the household worked as a CHW. The last criterion was included after the original protocol was drafted as at the time of the design the researchers were not aware that the CHW that would deliver the intervention resided in the villages selected for the study. It was explained that while all participating households would receive filters and stoves, half would receive them at the outset of the study and the balance at the conclusion of the 5-month follow-up period. After obtaining consent from the heads of participating households, a baseline survey was undertaken in September-November 2012 to collect information on demographics, socio-economic characteristics, water, hygiene and sanitation practices as well as fuel and cooking practices. Data collection tools were translated into *Kinyarwanda* and piloted before use.

Following the baseline survey, a public lottery was organised by the implementer and research teams during a village meeting to randomly allocate an approximately equal number of households from each village to intervention or control groups. Local authorities and village chiefs were extensively engaged to assess the suitability of this randomisation approach. After the lottery, members of control households were invited to leave the venue while those of intervention households attended a demonstration on the use and maintenance of the filter and stove, collected their devices, and carried them to their homes.

### Outcome assessment

#### Compliance

Monthly cross-sectional surveys were conducted by trained field investigators (the evaluation team) working independently of the implementation team at unannounced visits among each household. At each visit participants were asked to identify the main drinking water container in the household, whether it was the intervention filter or another container; the surveyor also recorded whether the filter contained water at the time of the visit, a possible objective indicator of filter use. The field investigators also observed the cookstove and if cooking was taking place at the time of the unannounced visit, recorded where and whether such cooking was on the intervention stove or the traditional stove. If no cooking was taking place, field investigators noted the presence of smoke marks on the intervention stove, a possible objective indicator of use. Reported measures of stove use were also collected by asking participants what stove had been used the last time cooking took place in their home.

Independent to our study, the implementers undertook a separate survey, conducted by Environmental Health Officers (EHOs), to assess use and acceptability of the intervention for their own monitoring and evaluation purposes. The details of this assessment have been presented elsewhere [23].

Additionally, to assess use of the intervention in a more objective manner, remotely reporting electronic sensors were mounted onto 23 intervention filters and 27 intervention stoves and deployed in a randomly selected sub-sample of intervention households for a two-week period. The details of the implementation of this nested study, data handling and analysis, and results are presented elsewhere [24].

#### Water quality

During each of the five monthly visits, field investigators took a sample from the water container identified by the householder as being used mainly for drinking by children under 5 years of age, or adults if no under 5 s resided in the household. If this was other than directly from the intervention filter, a second sample was taken directly from the filter if it contained water. All water samples were collected in sterile Whirl-Pak bags (Nasco, Fort Atkinson, WI) containing a tablet of sodium thiosulphate to neutralize any halogen disinfectant. Samples were placed on ice and processed within 6 h of collection to assess levels of TTC. Microbiological assessment was performed using the membrane filtration technique [25] on membrane lauryl sulphate medium (Oxoid Limited, Basingstoke, Hampshire, UK) using a DelAgua field incubator (Robens Institute, University of Surrey, Guilford, Surrey, UK).

#### Household air pollution

Monitoring of particulate matter with an aerodynamic diameter <2.5 µm (PM_2.5_) in the main cooking area took place between November 2012 and March 2013. 126 households (63 control and 63 intervention households) were randomly selected for semi-continuous 24-h PM_2.5_ monitoring. Households were numbered and selected by using a computerised random number generator. Upon arrival at the participant's home, the family member mainly responsible for cooking was identified and a short survey was employed to identify the area in the household where cooking primarily took place. “Stacking” of stoves (using different stoves, often in different locations) [26] was a common scenario, both in control and intervention households, though more common in the latter. In cases where the participant reported cooking equally in two locations or with two or more stoves, we sampled from indoor rather than outdoor locations and from traditional rather than intervention stove. UCB-PATS PM_2.5_ monitors (described below) were placed 1.5 m above the ground and 1 m away from the stove and, whenever possible, at least 1.5 m from windows and doors by suspending the monitors from the roof beams. When cooking was reported to take place outdoors, the PM_2.5_ monitor was mounted onto a vertical wooden stand and placed at the same distance and height from the stove. The location of the stand was marked on the floor and participants were advised not to touch or move the equipment.

PM_2.5_ was measured using the University of California, Berkeley Particle and Temperature Sensor (UCB-PATS™), (Berkeley Air Monitoring Group, USA), a semicontinuous (1-min averages), light-scattering nephelometer [27], [28]. Laboratory and field validations of the UCB-PATS have been described previously [27]–[30]. To take into account that nephelometer sensitivity is a function of an aerosol's specific optical properties such as size, colour, and shape [31], calibration of the UCB response with the target aerosol was undertaken by conducting 24-h PM_2.5_ gravimetric co-location measurements in a sub-sample of homes (n = 30). Five field blanks were obtained, resulting in an adjustment of subtracting 5 µg to the final filter masses (<1% of the mean mass deposition). The UCB-PATS response was then linearly regressed against the gravimetric samples (n = 27, R^2^ = 0.86), with the resulting equation then used to adjust the UCB-PATS response to the gravimetric measures (Figure S1 of supporting information). Three gravimetric samples were omitted due to incomplete sampling durations.

Gravimetric PM_2.5_ samples were collected using standard air sampling pumps (PXR8, SKC Inc., USA) with PM_2.5_ cyclones (SCC 1.062, BGI, USA) using a flow rate of 1.5 L/min. Flow rates were measured before and after installation of the sampling equipment in the home with a rotameter (Matheson Trigas, Montgomeryville, PA, USA) that had been calibrated using a TSI Flow Calibrator 4146 (TSI, Inc., USA). PM_2.5_ was collected on 37-mm Teflon filters (Pall, USA). Filters were stored at 4°C until shipment to Berkeley Air Monitoring Group in California, USA for weighing. Filters were equilibrated for 24 h at 22±3°C and 40±5% relative humidity before being weighed on a 0.1 microgram resolution electro microbalance (XP2U, Mettler Toledo, USA).

### Data analysis

All data were analysed using Stata 12 (Stata Corporation, College Station, TX, USA). Because both PM_2.5_ concentrations and TTC counts in drinking water followed non-normal distributions, medians, geometric means and Williams means are presented together with arithmetic means. The Williams mean is calculated by adding 1 to all the data values, then taking the geometric mean, then subtracting 1 again [32]. Categorical data were compared using a Chi square or a Fisher's exact test where appropriate. The non-parametric Wilcoxon rank sum test was used to compare PM_2.5_ concentrations in the main cooking area between intervention and control groups. To assess the effect of the intervention on water quality, TTC counts during follow-up were compared using random effects negative binomial regression as describe elsewhere [33] to account for (i) repeated observations within households and, (ii) the skewed distribution of the TTC counts. Model comparison was assessed by using the Bayesian information criterion (BIC), which is a well-established measure of goodness of fit that also applies to non-nested models [33], [34]. For the purpose of analysis, plates that yielded coliform forming units (CFUs) that were too numerous to count (TNTC) were assigned a value of 300 TTC/100 mL. Data were analysed in an intention-to-treat basis in order to estimate the effect of the intervention regardless of compliance. Only those households with complete follow-up data were analysed.

### Ethics

The study was reviewed and approved by the ethics committee at the London School of Hygiene and Tropical Medicine (No. 6239, as amended) and the Rwanda National Ethics Committee (No. 328 RNEC/2012). Written informed consent to participate in the research was obtained from the male or female head or the wife of each participating household.

## Results

### Study population

**Figure 1:**
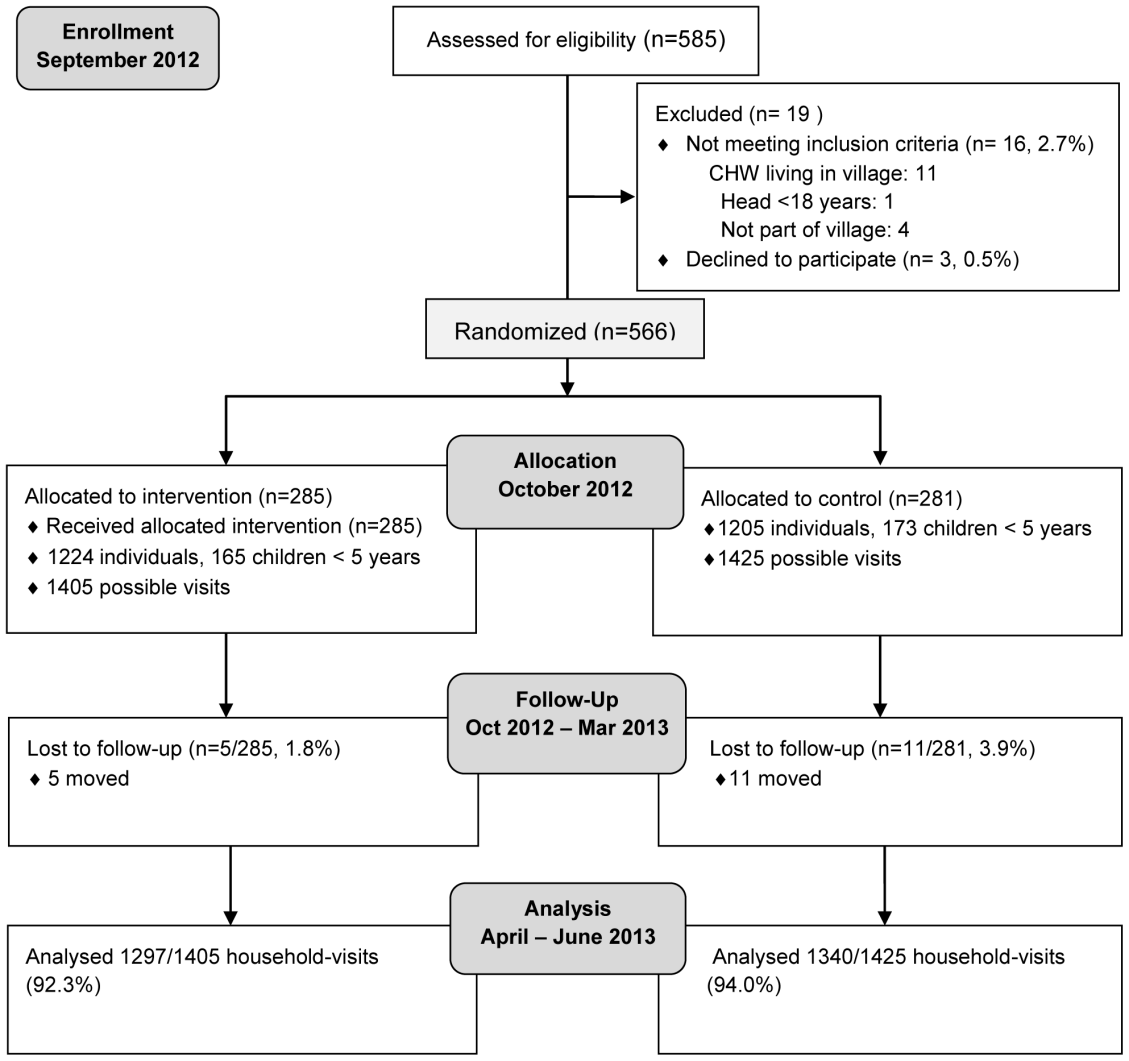
CONSORT diagram showing the flow of participants through the trial.

The three villages participating in the study comprised 585 households, all of which were screened to participate in the study, 16 (2.7%) were ineligible and 3 (0.5%) refused to participate (Figure 1). A total of 566 households with 2429 individuals were enrolled in the study. Of those 281 (49.7%) were assigned to the control group and 285 (50.4%) were assigned to receive the intervention filter and cookstove. Household loss-to-follow-up was 2.8%, primarily due to participants moving out of the study area. A total of 2737 household-visits were completed during the follow-up period (96.7%) and data on one of the primary outcomes (water quality) was collected for 2637 households-visits (93.2%).

### Baseline characteristics

Baseline characteristics were distributed evenly between the trial arms, with the exception of availability of soap among households with a designated hand washing area and boiling or chlorination of drinking water (see Table S1 of supporting information). At baseline, drinking water samples were obtained from 551 (97.3%) households. The median and Williams mean of drinking water was 14 and 20.2 TTC/100 mL (95% CI: 15.0–27.0 TTC/100 mL) and 22 and 30.3 TTC/100 mL (95% CI: 22.8–40.2 TTC/100 mL) for control and intervention groups, respectively.

### Filter and improved stove use and compliance

Most households used the filter throughout the study period (Table 1). Intervention households identified the filter as the main drinking source in 89.2% of all household visits where drinking water was available. Visual inspection at the time of the unannounced visit was consistent with reported use, with 99% of the filters containing water. Of the 10.8% of intervention households that stored their drinking water elsewhere, overall only 39.0% of them reported that the water had been treated with the intervention filter. Over the course of the study, however, only 62.9% of intervention households identified the filter as the main drinking water storage container in all five follow-up visits with available water (n = 240, 84.2%). Of the remainder, 11.2% reported treating it and storing it elsewhere at least once during the 5-month follow-up, 25.0% reported drinking untreated water at least once during follow-up and 0.8% did not know the status of their water in at least one of the visits. During the last follow-up visit, the major reasons for not having filtered water at the time of the visit were (i) forgetting to fill the filter (48.1%), (ii) drinking mainly locally produced beer instead of water (22.2%), or (iii) having a broken or not properly functioning filter (18.5%).

**Table 1:** Filter and stove use among intervention households: Evaluator's survey.

	All visits	
	N	%
**Filter use** 1		
Reported drinking container		
Intervention filter	1210	89.2
Other container	146	10.8
Water stored in other container treated	57	39.0
Method of treatment: Intervention filter	56	98.2
No water in intervention filter among households identifying filter as drinking container	12	1.0
No water in intervention filter among households not identifying filter as drinking container	83	56.8
**Stove use** 2		
**Observation data on use**		
Intervention household cooking at time of visit	280	26.9
Stove in current use		
Intervention stove only	152	54.3
Both stoves simultaneously	12	4.3
Traditional stove only	116	41.4
Currently cooking outdoors	59	21.1
Intervention stove users cooking outdoors^3^	49	32.2
**Reported data on use**		
Reported last stove used^4^		
Intervention stove only	593	78.0
Both stoves simultaneously	15	19.3
Traditional stove only	147	2.0
Reported using intervention stove in last three follow-up visits	130	47.5

1 Based on households that completed the visits and allowed enumerators to observe the container, 1356/1393 = 97.3%.

2 Data only available from mid follow-up 2 onwards (1040/1393 = 74.7%).

3 Among those households cooking only on the intervention stove.

4 Excludes those households cooking at time of home visit.

The intervention stoves were also used throughout the study, though most householders also continued to use their traditional stoves. Field investigators observed actual cooking on about a quarter (26.9%) of their unannounced visits. Of these, 54.3% were cooking only with the intervention stove and 4.3% were using both the intervention and traditional stoves (Table 1). Reported use was higher, with householders claiming they last cooked solely on the intervention stove on 78.0% of visits. Use of the intervention stove was not consistent, with 47.5% of intervention households reporting to have used the intervention stove during the last cooking event at all three home visits (data not collected during initial phases of follow-up). Likewise, of the households that were cooking at all three unannounced visits (n = 8), or at two of the three unannounced visits (n = 52), only 50.0% and 34.6% were using the intervention stove at all three or two visits, respectively. During the last follow-up visit, the major reasons for not using the intervention stove during the last cooking event were (i) having no time to tend the fire (34.1%), (ii) not having dry (30.7%) or the right-size wood (10.2%) or, (iii) cooking beans for which a traditional stoves was regarded most appropriate (10.2%).

Data on use of the intervention from the implementer's survey was very similar to our assessment (Table 2). A similar percentage of intervention households (27.7%) were cooking at the time of the visit. Of these, just over two thirds (64.1%) were exclusively cooking on the intervention stove, but only 17.2% of these were cooking outdoors, a figure just slightly lower than the one observed on our independent follow-up. Data from the implementer's more extensive survey confirmed the stacking of stoves in intervention households, with 76.4% of intervention households reporting to continue using their traditional stoves. Of these, 26.7% reported using it ≥7 times per week. Of interest was the fact that 83.8% of intervention households identifying the intervention stove as their primary cookstove reported that the intervention stove required more active tending of the fire as compared to the traditional stove.

**Table 2:** Filter and stove use among intervention households: Implementer's survey.

	N	%
**Filter use**		
Filter presence confirmed in households^1^	283	99.7
Tap accessible to <5 s	267	94.4
Water present in filter	269	95.1
**Stove use**		
**Observation data on use**		
Intervention household cooking at time of visit^2^	78	27.7
Stove in current use		
Intervention stove only	50	64.1
Both stoves simultaneously	4	5.1
Traditional stove only	24	30.8
Currently cooking outdoors	30	38.5
Intervention stove users cooking outdoors^3^	28	17.2
**Reported data on use**		
Reported last stove used^4^		
Intervention stove only	163	79.9
Both stoves simultaneously	2	1.0
Traditional stove only	39	19.1
Primary stove in current use is intervention stove^4^	253	89.1
Use intervention stove ≥7/week	236	93.3
Use intervention stove ≥14/week	137	54.2
Continue using traditional stove	217	76.4
Use traditional stove ≥7/week	58	26.7
Reported cooking less indoors	175	61.6
Reported main cooking is outdoors	163	57.4
Tend more the fire with the intervention stove^5^	212	83.8

1 Observation not allowed in one household.

2 Of those households that allowed the observation (n = 282, 99.3%).

3 Among those households cooking only on the intervention stove.

4 Excludes those households cooking at time of home visit.

5 Among those households identifying intervention stove as main cooking stove.

In the last round of our follow-up, 5.4% and 5.1% of intervention households reported having problems with their filter or stove at the time of the visit, respectively. Data collected from the implementer's repair team indicates that 24.9% of filters and 6.7% of stoves had to be repaired during the study. No devices had to be fully replaced, though some repairs involved the replacement of individual components. The main reasons for filters being repaired were (i) filters being clogged (48.6%) and, (ii) tubes being damaged by rodents (27.0%). The main reasons for the intervention stoves being repaired included (i) pot skirts melting (65%), and (ii) stick supports breaking (10%).

Overall, only 1.0% of water samples collected from control households were reported to have been treated with a neighbour's intervention filter, showing low levels of cross-contamination between groups.

### Water quality

**Figure 2:**
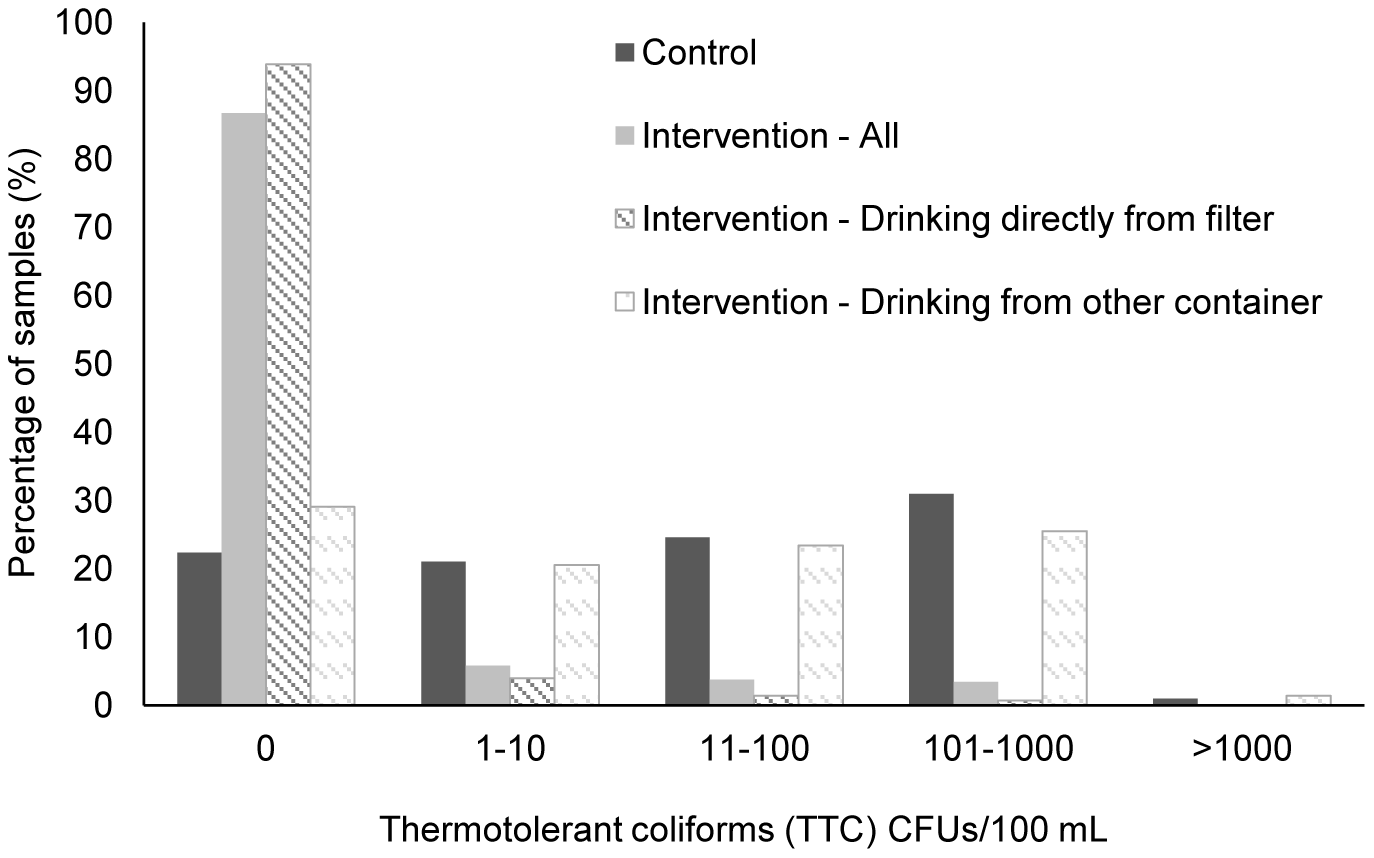
Percentage of water samples by level of contamination (TTC/100 mL).

The microbiological quality of the stored drinking water was significantly higher in intervention households than control households (Williams means 0.5 vs. 20.2 TTC/100 mL, respectively, *p*<0.001). Overall, 86.8% (95% CI: 84.9%–88.6%) of drinking water samples from intervention households were free of TTC compared to 22.4% (95% CI: 20.1%–24.6%) of control household samples (*p*<0.001) (Figure 2). The proportion of samples that had >100 TTC/100 mL was 3.6% (95% CI: 2.6%–4.6%) for intervention households and 31.9% (95% CI: 29.4%–34.5%) for control households. Overall, 96.6% of drinking water samples collected directly from filters were free of TTC. In intervention households, water quality was significantly higher in water samples collected directly from the filter (Williams mean 0.14 TTC/100 mL; 95% CI: 0.10–0.18) than water stored in another container (Williams mean 13.8 TTC/100 mL; 95% CI: 9.0–20.7) (see Table S2 of supporting information). The quality of the drinking water stored in other containers did not differ significantly between control and intervention households (*p* = 0.07). However, among intervention households, water that was stored in another container and was reportedly treated with the intervention filter was significantly of higher quality than reportedly non-treated stored water (Williams means 5.4 vs. 23.2 TTC/100 mL, respectively, *p*<0.001). Throughout the duration of the study, only 2.5% of control households had drinking water free of TTC on all follow-up visits as opposed to 56.5% of intervention households. Overall 15.2% of samples from control households and 5.1% of samples from intervention households yielded plates that were TNTC.

### Air quality

A total of 121 households (60 intervention and 61 control) completed the 24-h PM_2.5_ monitoring of the main cooking area. 66.7% of intervention households identified the intervention stove as their main cooking stove. However, only 23.3% of intervention households reported that their main cooking area was outdoors as promoted by the intervention. Of these, all households reported cooking with the intervention stove. Among the control households, the three stone fire was identified as the main cooking stove in 65.6% of cases, followed by the locally made *rondereza* stove (24.6%). Only one control household reported cooking outdoors.

Table 3 shows the PM_2.5_ concentrations of the main cooking area for control and intervention households on an aggregate level and stratified by reported main area of cooking. Overall, mean and median 24-h PM_2.5_ concentrations in intervention households were 0.485 mg/m^3^ and 0.267 mg/m^3^, respectively, compared to 0.905 mg/m^3^ and 0.509 mg/m^3^ for control households. This represents a 48% reduction in median 24-h concentrations (*p* = 0.005). Compared to control households that predominantly cooked indoors, intervention homes that reported indoor cooking showed a reduction in median concentrations of 37%, which was only borderline significant, possibly due to the smaller sample size (*p* = 0.08). Outdoor cooking in the intervention was associated with a median reduction of 73% when compared to control households (*p*<0.001) and 57% reduction when compared to indoor-cooking intervention homes (*p* = 0.02).

**Table 3:** Summary statistics for 24-h PM_2.5_ concentrations in the reported main cooking area.

PM_2.5_ (mg/m^3^)	N	Mean	SD	Min	Median	Max	Geometric mean	% Mean reduction	% Median reduction	Wilcoxon RST^1^ *p*-value
Control	61	0.905	1.05	0.06	0.509	4.69	0.51	-	-	
Intervention	60	0.485	0.53	0.04	0.267	2.28	0.28	46%	48%	0.005
Reported cooking location										
Control- Indoor cooking	60	0.910	1.06	0.06	0.506	4.69	0.51	-	-	
Intervention- Indoor cooking	46	0.558	0.56	0.04	0.321	2.28	0.33	39%	37%	0.08
Intervention- Outdoor cooking	14	0.243	0.34	0.05	0.139	1.40	0.16	73%	73%	<0.001
^1^Wilcoxon rank-sum (Mann-Whitney) test										

## Discussion

We report on a randomised controlled trial to independently evaluate a pilot implementation program distributing free water filters and improved cooking stoves to rural homes in Rwanda. We found high reported use of the intervention filter, which was associated with significantly higher microbiological quality of drinking water when consumed directly from the filter. Nevertheless, such use was not exclusive; a sizable proportion of householders continued to drink untreated water. We also found improved household air quality among intervention households despite continued use of the traditional stove.

Filter uptake among the intervention population was high, with filters being reportedly used in 89.2% of all household visits. Similar levels of uptake of filter-based interventions have been reported elsewhere [16], [35], [36]. Nevertheless, we found that 25% of intervention householders were reporting untreated water in at least one of the five follow-up visits. The nested study within this RCT using remotely reporting electronic sensors that collected objective data on use of the intervention devices (mainly times and volumes of water filtered for the intervention filter and times and duration of use for the intervention stove) corroborated our findings, showing that the filters and stoves were not used in a consistent and exclusive manner [24]. Epidemiological modelling based on quantitative microbial risk assessment suggests that even occasional consumption of untreated water can vitiate the health benefits associated with improved water quality interventions [37]–[39]. However, the intervention did significantly improve the microbiological quality of the drinking water when the filter was used as the main storage container. Since 96.6% of drinking water samples collected directly from filters were free of TTC, the conditions for achieving health gains may be achieved with better messaging.

Exclusive use was more problematic for the intervention stove. Only half of the intervention households reported that the last cooking event was performed with the intervention stove in the last three monthly follow-up visits. Likewise, only a third of those households that were visited twice at times that cooking was taking place were using the intervention stove at both instances, showing that among the intervention arm, households continued to rely on their traditional stove. Results from the implementers' survey showed similar results, with 76.4% of households reporting the continued use of their traditional stove, 26.7% of them using it more than 7 times per week. This is consistent with other studies that have shown that the introduction of a new stove often results in “stacking” rather than an immediate complete substitution [40]–[43].

Households reported continuing the use of their traditional stove because the intervention stove required more tending, unavailability of the adequate fuel or personal preferences for cooking traditional dishes. Context-specific issues regarding a community's cooking needs and preferences have been commonly cited in the literature as reasons for not achieving higher uptake and/or exclusive sustained use of improved cookstoves [42], [44]. Thus re-considerations of the promoted stove or more active messaging addressing each of the main barriers may be required if a switching of the stove as opposed to an addition of the intervention stove to the current cooking system is to be achieved. This is not only going to affect the potential health impact of the intervention but also its environmental impact.

The assessment of HAP among control and intervention households showed an overall reduction of 48% of 24-h PM_2.5_ among intervention households, which was comparable to reductions in household air pollution for rocket stove interventions in Ghana (52%) and Kenya (33%) [22], [45]. Indoor cooking with the intervention stoves as opposed to the traditional stove was associated with a 37% reduction in 24-h PM_2.5_, which was of borderline significance. However, we cannot rule out that this association may be due to residual bias by comparing sub-groups. Likewise, cooking outdoors, as recommended by the implementer, doubled the reduction in 24-h PM_2.5_ from 37% to 73% as compared to indoor cooking on traditional stoves. Future studies, randomising participants not only to stove technology but also to cooking location (indoors *vs*. outdoors) would be advisable. More effective messaging may increase the levels of outdoor cooking expected by the intervention, as only 57.4% of households reported that their main cooking area was outdoors. Nevertheless, both the indoor and outdoor concentrations in the cooking area were well over even the initial interim 24-h WHO target for PM_2.5_ (75 µg/m^3^) [46]. At the same time, it will be important to monitor personal exposure directly, as most householders that identified the intervention stove as their primary cooking stove (83.8%) reported that the intervention stove required more tending than their traditional one, which could mitigate some of the impact from the household level reductions in PM_2.5_. Indeed, many studies have found that reductions in personal exposure tend to be lower than reductions of emissions in the cooking area [47], [48]. Given that a recent RCT study suggested that personal exposure reductions exceeding 50% may be required to achieve meaningful health impacts [47], further assessments of the intervention stove maybe be needed to determine whether the use of the intervention stove translates into meaningful health benefits.

This study has certain limitations. First, the villages included in the RCT were not selected randomly and should not be viewed as representative of any larger population. Second, we cannot rule out the potential for reactivity due to repeated monthly follow-up visits [49]. Third, while we attempted to collect objective indicators of use, by both undertaking visual observations of the filter and stoves and cooking events, the study relied heavily on reported data, which is susceptible to reporting bias. Furthermore, in this study we failed to collect data on reported supplementation of treated water with untreated water, which would have further implications for the health impact of the study. Previous studies with the earlier version of the LifeStraw Family filter have found quite varied results. A study in the Democratic Republic of Congo showed substantial supplementation despite high levels of filter use [16]. On the other hand, a study among HIV-positive mothers, who may be more aware of their health and their children's health, reported almost no supplementation [36]. However, in the latter storage containers were provided. Fourth, budget constraints allowed only the main cooking area, as identified by the participant, to be monitored for HAP. Given the potential for reporting bias and that stacking was commonly reported among the study population, it is very likely that cooking events may have taken place during the monitoring period in areas other than the one being monitored, thus giving a misleading and probable underestimate of the actual total HAP. Likewise, budget constraints did not permit personal PM_2.5_ assessment, a more reliable metric for exposures associated with health outcomes [50]. Fifth, we did not collect any self-reported or other measures of diarrhoea or respiratory infections in our study communities. Finally, the follow-up period of this evaluation was limited to 5 months. This represents under a fraction of the lifespan of both the filter and stove and provided little opportunity to assess the impact of seasonal variations that are common in water quality and HAP. It also provided no opportunity to assess long-term patterns of use, which have been shown to diminish or vary over time for both water filters and improved cookstoves [35], [43]. We are endeavouring to address some of these shortcomings in a longer-term follow-up study, currently underway, that will focus on health outcomes and sustained use.

Notwithstanding these limitations, this study suggests that a combined filter/stove intervention accompanied by consistent follow-up to promote use has the potential to significantly improve drinking water quality and household air pollution among a vulnerable population in Rwanda. If the longer-term follow-up study demonstrates sustained use with more exclusive reliance on the intervention hardware and lower personal exposure to HAP, then a large-scale roll out in Rwanda could significantly reduce exposures linked to much of the country's disease burden.

## Supporting Information

Figure S1 **Mass calibration of UCB-PATS against co-located PM_2.5_ gravimetric samples.**
(TIF)

Table S1 **Baseline characteristics of participating.**
(XLSX)

Table S2 **Summary statistic of TTC/100 mL in household drinking water and filter effluent by study group.**
(XLSX)

Text S1 **Protocol.**
(DOC)

Text S2 **CONSORT Checklist.**
(DOCX)
