# Supplementary figures and images for: Assessing the Impact of Water Filters and Improved Cook Stoves on Drinking Water Quality and Household Air Pollution: A Randomised Controlled Trial in Rwanda
Source: PLoS One. 2014 Mar 10;9(3):e91011. doi: 10.1371/journal.pone.0091011 (PMC3948730; doi:10.1371/journal.pone.0091011)

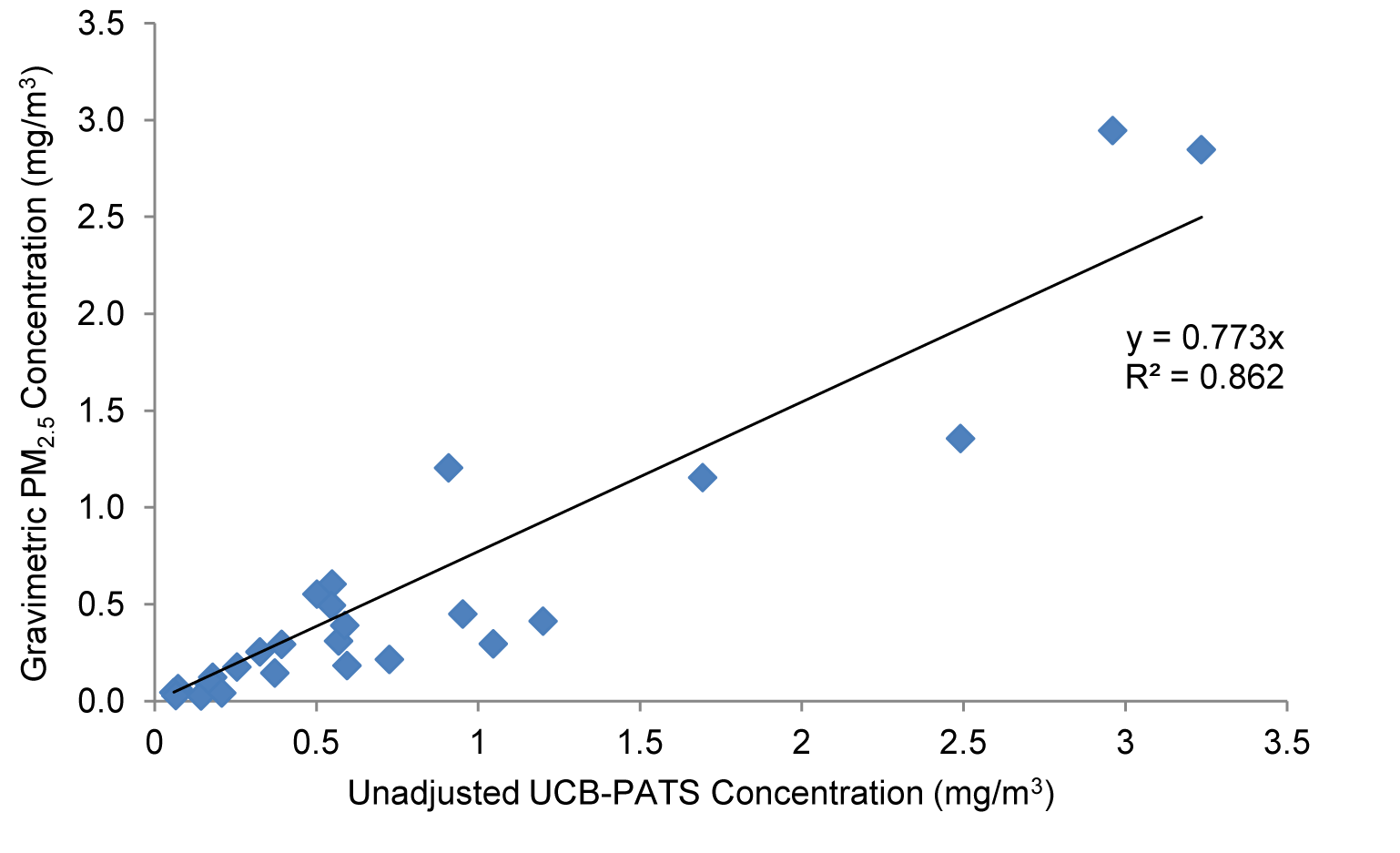

Supplement: Figure S1 — Mass calibration of UCB-PATS against co-located PM2.5 gravimetric samples. (TIF) [file pone.0091011.s001.tif]
