# Supplementary material for: Assessing the Impact of Water Filters and Improved Cook Stoves on Drinking Water Quality and Household Air Pollution: A Randomised Controlled Trial in Rwanda
Source: PLoS One. 2014 Mar 10;9(3):e91011. doi: 10.1371/journal.pone.0091011 (PMC3948730; doi:10.1371/journal.pone.0091011)
Supplement: Text S1 — Protocol. (DOC) [file pone.0091011.s004.doc]

STUDY PROTOCOL

ASSESSING A PILOT PROJECT TO DISTRIBUTE WATER FILTERS AND IMPROVED COOKSTOVES IN WESTERN RWANDA

1. SUMMARY

The London School of Hygiene & Tropical Medicine (LSHTM) has been engaged by Portland State University (PSU to conduct an independent evaluation to assess the uptake, performance and environmental exposure impact (drinking water and indoor air quality) of a pilot project in Rwanda to distribute water filters and improved cook stoves to 2000 households in 11 districts. The pilot project is designed to provide information on whether to proceed with a large-scale delivery of the intervention in Western Rwanda. The intervention is being implemented by DelAgua Health (DelAgua) in cooperation with the Rwanda Ministry of Health (MoH).

2. BACKGROUND

Environmental contamination at the household level is a major cause of death and disease, particularly among rural populations in low-income countries. Unsafe drinking water, together with poor sanitation and hygiene, account for an estimated 9.1% of the global burden of disease and 6.3% of all deaths. Much of this disease burden is associated with diarrhea, which alone accounts for 21% of deaths in children under 5 years in low-income countries. Indoor air pollution contributes to acute lower respiratory infection (ALRI), the leading cause death in children. These environmental hazards are further aggravated among rural inhabitants of sub-Saharan Africa who are more likely to rely on unsafe water supplies and cook using biomass fuels on inefficient stoves.

Inefficient stoves also present substantial economic, developmental and environmental costs. At the household level, poverty is exacerbated and time spent at school reduced by the burden of collecting up to 50% more fuel for boiling drinking water and cooking. Individuals, households and governments bear the cost of expenditures for seeking treatment of enteric and respiratory infections. Low-efficiency stoves contribute to denuding of forests for fuels, urban air pollution and greenhouse gas emissions.

With a population expected to reach 12 million in 2012, Rwanda is the most densely populated country in Africa. Eighty per cent of the population of Rwanda lives in rural areas and is engaged in agriculture. Despite significant progress over the last decade, 57% of the population is living below the poverty line, 37% of them living in extreme poverty. Annual per capita income increased from US$ 235 to US$ 291 between 2002 and 2008. Morbidity and mortality is still dominated by communicable diseases, which account for more than 90% of visits to health facilities. The most common communicable diseases are malaria, HIV/AIDS, acute respiratory infections, diarrhoeal diseases and tuberculosis.

In an effort to reduce the disease burden in rural Rwanda, decrease poverty associated with expenditures for fuel, and minimize the impact of greenhouse gases from inefficient combustion of biomass, the Rwanda Ministry of Health (MoH) and the Rwanda Environmental Management Authority (REMA) has partnered with DelAgua Health and Development (DelAgua) and Manna Energy (Manna) to design, deploy and evaluate the impact of an project that will deliver and promote the use of advanced water filters and high efficiency cook stoves to serve 3 million people in Western Rwanda. The intervention will be delivered by community health workers (CHWs) under the supervision of the MoH. It will be financed by carbon credits under the United Nations Clean Development Mechanism.

Prior to initiating the full campaign mid 2013, the implementers are undertaking a pilot distribution of 2000 filters and cook stoves. The objective of the pilot is to optimize delivery strategies and ensure that the accompanying promotional activities succeed in yielding high uptake and use of the hardware. The implementers have contracted with PSU to conduct the overall evaluation of the pilot. PSU, in turn, has engaged LSHTM to evaluate certain aspects of the pilot, namely the process for delivering the intervention, uptake and use of the hardware, and the impact of the intervention on drinking water quality and indoor air quality. These will be assessed by undertaking a process evaluation study and a separate randomized control trial (RCT). This protocol concerns only the RCT. Any evaluation of the full-scale roll out of the intervention will be subject to a separate protocol and ethics review.

3. STUDY OBJECTIVES

(i) To asses uptake and use of the intervention by the target population when delivered programmatically, and

(ii) To assess the impact of the intervention on the microbiological quality of household drinking water and air quality near the self-reported cooking area over the 5-month follow-up period.

4. INTERVENTION

The intervention consists of the delivery of advanced microbiological water purifiers and high efficiency cook stoves (described below) and promotion of their correct, consistent use. The intervention is delivered at the community level by CHWs who are engaged by the MoH to provide a variety of health related services to rural villages in Rwanda. The strategy and specific promotional activities for delivering the intervention were developed based on a pre-pilot project among 100 villages by the implementing organizations and approved by the MoH. Following informed consent from participating householders, CHWs will provide the intervention hardware and instructions for use. They will return to each household within 10-20 days following the initial delivery to answer questions and address any other issues. Thereafter, they will continue to visit participating households on a monthly basis for 5 months to provide further advice and collect information on acceptability and use of the hardware.

The LifeStraw Family Filter (LSF) 2.0 is a second-generation of gravity-based water purifier that incorporates a chamber for safely storing treated water. The microbial barrier consists of a hollow-fibre filter that has been shown to meet the highest standards for microbiological performance and with regular maintenance and use to last for more than 18,000L (an estimated 3 years for a family of 5) (Clasen 2009). The first generation LSF has been shown in field studies to be highly effective in improving water quality and consistent (though not exclusive) use (Boisson 2010). However, the previous generation (hanging) model relied on householders to either treat their water every time they wanted to drink or to provide their own device to protect water from recontamination in the home. By integrating a safe storage vessel within the filter itself, and by reworking the device into a table-top design, the second generation device to be used in Rwanda is expected to reduce recontamination and increase use.

The Eco Zoom stove is based on the 'rocket' concept that uses an internal 'chimney' in the stove that directs air through the burning fuel (usually biomass), and encourages the mixing of gases and flame above it. Precise internal stove dimensions are used to achieve high combustion efficiency and transfer heat to the cooking pot. Comparison of improved cook stoves for USAID by the Berkeley Air Monitoring Group in Haiti, Uganda and Tanzania reported that the EcoZoom (aka StoveTek) stove saved 39% to 54% of fuel compared to open fires, cooked meals faster, and was the most preferred stove by 96% of households (USAID 2011). To date, however, the Eco Zoom filters have not been deployed widely in Rwanda. Studies suggest that the health, economic and environmental benefits of these technologies are most affected by their relative fuel costs, time and fuel use efficiencies, the incidence and cost-of-illness of acute respiratory illness, and the cost of household cooking time (Ezzati 2001; Smith 2000). The actual impact of the stoves on indoor air pollution and health will depend, among other things, on fuels used, cooking practices, quantities and other sources of indoor air pollution, time spent in close proximity to stoves, etc. (Smith 2011).

5. STUDY DESIGN

A household randomized controlled trial (RCT) to assess the impact of the intervention on drinking water quality and indoor air quality. These are discussed more fully in Sections 6-9 below.

6. STUDY SITES; STUDY POPULATION

The pilot involves the distribution of filters and cook stoves to all participating households in 2 or 3 villages in each of 11 designated Districts in Rwanda: Rutsiro, Karongi, Ngororero, Nyamasheke, Nyabihu Districts of Western Province (excluding part of the district to the north of the Ruhengeri road); Rubuvu District (excluding part of the district to the north of the Ruhengeri road); Nyaruguru, Nyamagabe, Muhanga, and Ruhango Districts in Southern Province; Gakenke District in Northern Province. These Districts have been identified as areas of need by MoH for improvements in drinking water and fuel conservation. With an average of 100 households per village, the pilot is designed to cover a total of 2000 households. Households will be eligible to participate in the study if they reside in selected villages and are willing to participate after receiving complete details regarding the study.

The proposed RCT will be limited to Karongi and Ngororero districts of Western Province selected for their proximity to Kigali. In order to conduct the RCT, DelAgua will recruit an additional 200 households from the overall pilot population in these two districts. A total of 400 households will participate in the RCT. Following a baseline survey, the households that agree to participate in the RCT after receiving complete details will be randomly allocated to either receive the intervention at the outset of the study (intervention group) or 5 months later after completion of follow up (control group).

9. Eligibility, Enrollment and Baseline; Sample Size.

In Karongi and Ngororero districts of Western Province, 400 households will be recruited to participate in a randomized, controlled trial (RCT) to assess the impact of the intervention on drinking water quality and indoor air quality. Households will be eligible to participate in the RCT if they meet the selection criteria for inclusion in the pilot rollout of the intervention (i.e., reside in a household in a village selected for the pilot) and agree to participate after receiving complete details in accordance with a prescribed information sheet.

Each household will be assigned a unique identification number for collecting and recording household data; no household members will be identified by name. Enrollment procedure and data collection will be administered by enumerators trained in research ethics and data collection methods by LSHTM staff. After a baseline survey that includes demographics, water management and cooking practices, half of the RCT households will be randomly allocated to an intervention group that will receive filters and stoves as part of the pilot. The remaining 200 households will be asked to continue their existing water management and cooking practices and will receive filters and stoves at the end of the 5-month follow-up period. The method of random allocation will be decided once context-specific data is gathered by speaking to key informants (i.e. village chief, MoH workers, villagers etc.).

The sample size for the RCT is based on detection of CO_2_ emissions from the improved cook stove as derived from previous field studies (USAID 2011). In order to observe a minimum of a 15% reduction in CO2 emissions (power=80%, p=5%), the intervention and control arms should have 173 households per arm. 200 households will be recruited per study arm in order to allow for any early termination, such as a household relocating to another Sector, or voluntarily leaving the study. Reassessment of the sample size will be conducted during the first months of data collection.

9.2 Follow-up Sampling and Surveillance.

Each RCT household will be visited (i) after initial community-wide delivery of the intervention, (ii) once 10-20 days after delivery of the intervention to address any issues, and (iii) thereafter each month for 5 months, giving a total of 7 household visits over the course of 6 months. During the monthly follow-up visits, the trained CHW will conduct a short survey addressing use and acceptability of the filter and cook stove. Once during the 6 month period, trained Environmental Health Officers (EHOs) will conduct a separate survey to (i) assess water management and cooking practices and (ii) record specific observations about the use of the filter and cook stove. These visits are conducted under the implementer’s direction for their own monitoring and evaluation purposes.

Independently, LSHTM-trained staff will conduct their own enrollment and baseline survey and will conduct monthly follow-up visits for the following five months to (i) uptake and consistent use, (ii) collect water samples from stored drinking water in the home. In addition, for those households randomly selected for air quality monitoring, UCB monitors will be placed in the participant’s home for 24 consecutive hours. Water samples will be assayed for thermotolerant coliforms (TTC), a WHO-prescribed indicator of faecal contamination. Air quality monitoring will assess the concentration of PM_2.5_ in the main cooking area as identified by the household.

10. MEAUREMENT OF OUTCOMES

Outcomes of interest will be measured via a mixture of (i) survey questionnaire responses given by the household, (ii) observations made by enumerators, (iii) analysis of water samples and (iv) readings from instruments placed in the cooking area of participants for air quality.

The primary outcomes of this study are (i) to assess levels of faecal contamination (measured by thermotolerant coliforms, TTC) in stored water in the home that householders used for drinking, and (ii) to determine average 24-h concentrations of PM_2.5_ in the main cooking area as identified by participants. The secondary outcome of the study will be to assess the use of the filters and stoves based on self-report and spot-check observations.

11. DATA ENTRY AND MANAGEMENT; DATA ANALYSIS

11.1 Data Entry and Management. Data will be collected using prescribed survey tools on password-protected smartphones, encrypted and transmitted securely to a password protected database.

11.2 Data Analysis. The data will be validated by checking for completeness, consistency, and data recording errors. Stata Version 11.0 (Stata Corporation, College Station, Texas, US) will be used for analysis, including comparisons of intervention and control households using the t-test and the Wilcoxon test (where appropriate) for continuous outcomes, and the Fisher’s exact test or the Chi-square test for binary outcomes. Generalized estimating equations or random effects models will be used for the analysis of binary outcomes to control for repeated measurements at the same household. Statistical analyses of microbiological data are conducted after log_10_ transformation of TTC count values to account for the skewed distribution.

12. ETHICS APPROVAL AND INFORMED CONSENT; CONFIDENTIALITY; POTENTIAL BENEFITS

12.1 Ethics, Risks and Informed Consent. This study will be submitted for approval by the Rwanda National Ethics Committee and the Ethics Committee of LSHTM. Informed consent will be obtained from all study participants according to the requirements of the Rwanda National Ethics Committee and the Ethics Committee of LSHTM. Risks to the study population are minimal. The filter medium and stoves have been fully assessed for safety and performance in the laboratory and are expected to perform in accordance with specifications, and have been certified by the Rwanda Bureau of Standards. While this is expected to improve drinking water quality and indoor air quality, the study population will not be put at risk even if the hardware does not perform since such hardware does not increase exposure. Participation is completely voluntary, and householders may withdraw at any time. No financial or other incentives will be offered or given to participants to solicit their participation.

12.2 Confidentiality. Questionnaires will be given a numerical unique identifier so that no personal identifiers such as names or locations of household will be included in program reporting. Raw data will be stored in a password-protected environment. Any records linking names to unique identifiers will be destroyed upon completion of data collection. Analytical datasets and all results presented and published will be de-identified at the household, village, cell, sector and district level.

12.3 Potential Benefits. Potential benefits to subjects include the improvements in drinking water quality, indoor air quality, and time- and cost-savings associated with using less fuel. If the results of the pilot rollout show the intervention to be effective, then the full scale campaign will be undertaken (subject to certain conditions) using information from this RCT sudy and the larger evaluation to optimize the distribution and maximize coverage and uptake. This creates the potential for millions of Rwandans to benefit from the intervention.

References

Boisson S, Kiyombo M, Sthreshley L, Tumba S, Makambo J, Clasen T (2010). Field assessment of a novel household-based water filtration device: a randomised, placebo-controlled trial in the Democratic Republic of Congo. PLOS One 5(9): e12613. doi:10.1371/journal.pone.0012613

Clasen T, Naranjo J, Frauchiger D, Gerba C (2009). Laboratory assessment of a gravity-fed ultrafiltraton water treatment device designed for household use in low-income settings. Am J. Trop. Med. Hyg. 80(5) 819-823

Ezzati M, Kammen DM. Quantifying the effects of exposure to indoor air pollution from biomass combustion on acute respiratory infections in developing countries. Environ Health Perspect. 2001 May;109(5):481-8

Smith KR. National burden of disease in India from indoor air pollution. Proc Natl Acad Sci U S A. 2000 Nov 21;97(24):13286-93.

Smith KR, McCracken JP, Weber MW, Hubbard A, Jenny A, Thompson LM, Balmes J, Diaz A, Arana B, Bruce N (2011). Effect of reduction in household air pollution on childhood pneumonia in Guatemala (RESPIRE): a randomised controlled trial. Lancet. 12;378(9804):1717-26

Rwanda Ministry of Finance and Economic Planning, Economic Development and

Poverty Reduction Strategy. 2008.

USAID.  2011. In-home emissions of greenhouse pollutants from rocket and traditional biomass cooking stoves in uganda. <http://transition.usaid.gov/our_work/economic_growth_and_trade/energy/publications/uganda_emissions_report.pdf>
